# Two-Week Protocol Biopsy in Renal Allograft: Feasibility, Safety, and Outcomes

**DOI:** 10.3390/jcm11030785

**Published:** 2022-01-31

**Authors:** Manuel Lim, Byung Kwan Park, Kyo Won Lee, Jae Berm Park, Kyeong Deok Kim, Jaehun Yang, Jieun Kwon, Eun Sung Jeong, Seunghwan Lee

**Affiliations:** 1 Department of Surgery, Samsung Medical Center, Sungkyunkwan University School of Medicine, Seoul 06351, Korea; ykcywbd@gmail.com (M.L.); jaeberm.park@samsung.com (J.B.P.); kdkim7438@gmail.com (K.D.K.); gsyangjh@gmail.com (J.Y.); kje0426@gmail.com (J.K.); asurada1405@gmail.com (E.S.J.); 2 Department of Radiology, Samsung Medical Center, Sungkyunkwan University School of Medicine, Seoul 06351, Korea; 3 Department of Surgery, Kyung Hee University Hospital at Gangdong, Seoul 05278, Korea; histones@hanmail.net

**Keywords:** kidney transplantation, protocol biopsy, complication

## Abstract

Background: Protocol biopsy in renal allograft helps to early detect subclinical rejection (SCR) in patients who have no abnormal clinical and laboratory findings. Still, there are rare reports about the techniques and outcomes of two-week protocol biopsy. The aim of this study was to assess two-week protocol biopsy regarding the technical feasibility, procedure safety, and clinical outcomes. Methods: A total of 894 protocol biopsies were performed in adult recipients between 2012 and 2019. Two-week and one-year protocol biopsies were guided with ultrasound in 842 and 399 patients by one of four radiologists with wide range of biopsy experience, respectively. These protocol biopsies were compared in terms of feasibility and safety. Standard references were clinico-laboratory findings and biopsy examinations. Results: The median period of two-week and one-year protocol biopsies were 12 days (10–20 days) and 383 days (302–420 days), respectively. All protocol biopsies were technically successful and there was no difference between radiologists regarding technical success and complications (*p =* 0.453). Major complication (Clavien–Dindo grading II–IV) rates of two-week and one-year protocol biopsies were 0.3% (3/842) and 0.2% (1/399), respectively (*p =* 1.000). However, univariate analysis demonstrated that platelet count < 100 K/mL and blood urea nitrogen ≥ 40 mg/dL were associated with major complications in two-week protocol biopsy. The SCRs of these protocol biopsies were 15.4% (130/842) and 33.6% (134/399), respectively (*p <* 0.001). Conclusion: Two-week protocol biopsy is technically feasible and safe. It contributes to early detecting a substantial number of SCRs. Prior to the biopsy, platelet count and blood urea nitrogen should be carefully checked to predict major complications.

## 1. Introduction

Percutaneous biopsy is accepted as a gold standard for identifying the cause of renal allograft dysfunction [1]. It is useful for detecting subclinical rejection (SCR) as well as several pathological conditions such as de novo or recurrent glomerulonephritis, BK virus related nephritis, and calcineurin inhibitor nephrotoxicity [2,3]. Traditionally, renal allograft biopsy has been recommended in patients who have changes in clinical conditions and abnormal biochemical tests. However, when the clinical diagnosis is histologically confirmed, the degree of renal damage tends to be so advanced that treatments cannot be so effective [4]. For many years, several researchers have reported that acute rejection is revealed at an early stage with protocol biopsy and that early detection and treatment of SCR improves clinical outcomes before renal dysfunction [5,6,7,8]. When protocol biopsy was first introduced, many clinicians were reluctant to perform it on stable transplants due to fear of possible complications, arguing that the risks were ethically unjustified [9]. However, several groups have reported that renal allograft biopsies under ultrasound (US) guidance had very low complications [9,10,11]. Currently, early protocol biopsy becomes more available to early detect SCR.

Still, there is no consensus on when protocol biopsy is appropriate. Reportedly, early protocol biopsy was conducted over a period of 1 month to 6 months [12,13,14,15]. It may run the higher risk of bleeding complications than late protocol biopsy if renal allograft is not yet settled down. Post-operative fibrosis cannot be completed around the renal allograft within post-transplant one month. We had experienced many cases of two-week and one-year protocol biopsies to early detect SCR. Still, there are rare studies reporting the utility of two-week protocol biopsy. Therefore, it was hypothesized that two-week biopsy can be performed feasibly and safely and that it can detect early SCR. The purpose of this study was to assess the technical feasibility and clinical outcomes of two-week protocol biopsy in patients without abnormal clinical and biochemical findings.

## 2. Materials and Methods

### 2.1. Study Population and Design

**Figure 1:**
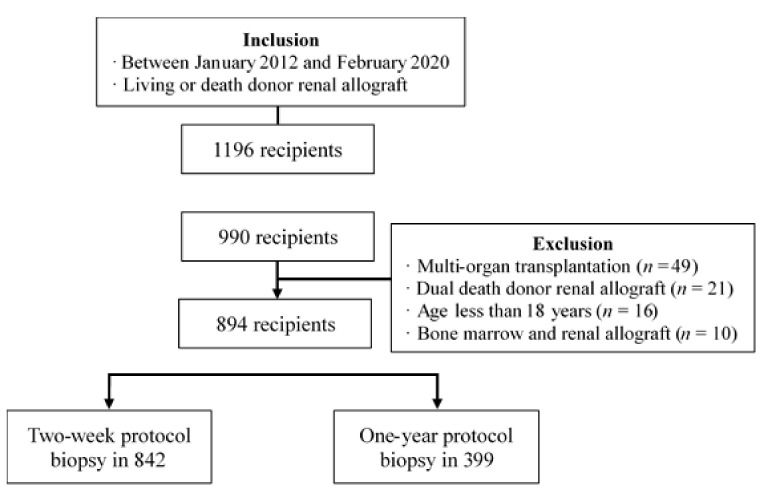
Flow diagram of study population.

This retrospective study was conducted from January 2012 to February 2020 in 1196 adult patients who underwent kidney transplantation (KT) in a single center. This study was approved by the Institutional Review Board (File No. 2020-04-110-001). The inclusion criteria included patients who underwent living or deceased donor KT during the period and who underwent two-week or one-year protocol biopsy. A total of 990 patients satisfied the inclusion criteria. Among these patients, 96 were excluded according to the exclusion criteria including multi-organ transplantation (*n* = 49), dual KT (*n* = 21), under 18 years (*n* = 16), and combined kidney-bone marrow transplantation (*n* = 10) [16]. Finally, 894 patients were included for analysis. Among these patients, Two-week and one-year protocol biopsies were performed in 842 and 399, respectively (Figure 1).

### 2.2. Protocol Biopsy Procedures

Early and late protocol biopsies was performed at around two weeks (median, 12 days; range 10–20 days) and one year (383 days; 302–420 days), respectively. Anticoagulant and antiplatelet agents were discontinued at least 7 days prior to biopsy. In cases where it was difficult to stop anticoagulant and antiplatelet agents, the protocol biopsy was not performed.

One of four genitourinary radiologists performed the protocol biopsies. Their experience of renal allograft biopsy ranged 1 month–10 years. Biopsies were guided with ultrasound (IU22, Philips Healthcare, Best, The Netherlands) in the department of radiology. A 3 MHz convex probe (Philips Healthcare) was used to localize an allograft. After controlling gray-scale and Doppler scale parameters, we assessed the allograft size, cortical echogenicity, collecting system, and fluid collection. The cortex to be biopsied was determined where it was most safely accessible. Biopsy cores were sampled with free hand techniques and a biopsy guider was not set on the probe. An 18-gauge semi-automated needle was used for sampling. Manual compression following biopsy was a routine procedure to minimize bleeding and post-compression US was additionally performed to determine if bleeding was present. When bleeding was detected despite manual compression, we continued to compress the biopsy site until it disappeared.

### 2.3. Immunosuppressive Protocol

For induction, basiliximab (Simulect, Novartis Pharmaceuticals, Basel, Switzerland) was usually administered, and rabbit antithymocyte globulin (rATG) (Thymoglobulin, Genzyme, Cambridge, MA, USA) was administered for several indications. In the living donor case, in positive human leukocyte antigen (HLA) crossmatch, in the donor-specific antigen (DSA) with a mean fluorescence intensity ≥2500, and in the ABO-incompatible case, the rATG was administered to the patients with 1.5 mg/kg/kg on day 0 and postoperative days 1 and 2. In the case of deceased donor, rATG was administered in the extended criteria donor KT. In all other cases, basiliximab was administered, at a dose of 20 mg/day, on the 0 and on postoperative day 4.

For maintenance, all patients were treated with a triple immunosuppressive regimen of tacrolimus, mycophenolate mofetil, and methylprednisolone. Tacrolimus (FK506, Prograf; Astellas Fujisawa, Osaka, Japan, and generic tacrolimus) was started at 0.1–0.15 mg/kg/day in the afternoon on the day of surgery and was administered twice a day. The blood trough level was maintained at 8–10 ng/mL until one month after surgery and at 5–8 ng/mL thereafter. Mycophenolate mofetil (Myfortic; Novartis Pharma AG, Basel, Switzerland) was started with tacrolimus at 540 mg twice daily. Methylprednisolone was started on the day of surgery at an intravenous dose of 500 mg/day and administered for 2 days and then tapered by half every day to 60 mg/day. Oral methylprednisolone was administered at 32 mg/day for 7 days, 16 mg/day for the next 2 weeks, 8 mg/day for the next month, and 4 mg/day for maintenance. Methylprednisolone withdrawal was attempted for patients with low immunologic risk (DSA-negative and ABO-compatible) and without acute rejection episodes within 3 months after KT. Post-transplant steroids were gradually tapered off and totally withdrawn more than 6 months after KT.

In the case of immunologic risk group (Positive HLA crossmatching, positive DSA) and ABO incompatible KT, a desensitization protocol was applied. Monoclonal antibody against CD20 (Rituximab; Genentech, Inc., South San Francisco, CA, USA) at 375 mg/m^2^ or 200 mg was administered one month before transplantation. Plasmapheresis (PP) was started on the following day, and was performed 5 times. Intravenous immunoglobulin (IVIG) at 400 mg/kg was administered after every PP session. The rATG was administered for induction agent on day 0 and postoperative days 1 and 2. For ABO-incompatible KT, PP frequency depended on baseline anti-ABO titer and target titer before transplantation.

### 2.4. Definition and Treatment of SCR

Biopsy cores were assessed using Banff 2007 classification and evaluated by a pathologist who had more than 10-year experience in urologic pathology. Specimens were embedded in paraffin, stained with H&E and PAS, and checked with immunohistochemistry for C4d and SV40. He assessed borderline changes, acute cellular rejection, and antibody-mediated rejection in the treatment range of SCR. When patients were diagnosed with acute cellular rejection and borderline change, steroid pulse therapy was administered with an intravenous methylprednisolone dose of 500 mg/day for 3 days, which was then tapered by half every day to 60 mg/day. Hence, oral methylprednisolone was initiated at 32 mg/day after intravenous administration and then tapered to 4–8 mg/day within 1–2 weeks and to 4 mg/day for maintenance. When patients were diagnosed with antibody-mediated rejection, PP and IVIG were administered according to the protocol. Treatment for SCR was not mandatory and was decided in consideration of the patient’s underlying condition, clinical course, immunological risk, and infection risk.

### 2.5. Data Analysis

Two-week and one-year protocol biopsies were compared in terms of technical feasibility such as technical success, number of cores, number of glomeruli, length of core, allograft location, and biopsy duration. Technical success of the protocol biopsies was defined if more than 20 glomeruli were sampled [17,18,19,20]. Our pathologists asked us to sample 20 glomeruli or more because they needed to perform the three types of staining for light microscopy, electron microscopy, and immunofluorescence microscopy. Accordingly, we defined inadequate biopsy when the number of glomeruli was less than 20. Recipient’s age, sex, body mass index, comorbidities, induction of immunosuppressive agent, immunological risk factors, donor types, pre-biopsy laboratory results, rejection event, complications after biopsy, core and glomeruli count of biopsy specimen were reviewed and compared between the protocol biopsies. Complications were counted within 3 days after biopsy and analyzed according to Clavien–Dindo classification and radiologic examination. Clavien–Dindo classification is divided into 5 grades of severity according to the treatment of complications [21]. Major and minor complications were divided based on whether the problem resolved spontaneously without intervention [3]. Major complication was defined by Clavien–Dindo classification II-V requiring transfusion, intervention, or intensive care unit management [21]. Patient demographics, biopsy-related complications, SCR, and acute rejection were investigated by reviewing electronic medical charts.

### 2.6. Statistical Analysis

Data are expressed as mean ± standard deviation for continuous variables and as frequency (percent) for categorical variables. Patient demographics, complication and rejection results of protocol biopsies were compared by Mann–Whitney U test for continuous variables or Chi-square test for categorical variables. Univariate and multivariate logistic regression models were used to access the risk factors for major complication for two-week protocol biopsies. However, if it was impossible to select variables and build models because of rare complications, a non-inferiority test was used to compare the major complications of the protocol biopsies. According to the previously reported major complication rate of 0.4–1.0% [22], a margin of 1% was clinically set through a non-inferiority test, and the complication rates of protocol biopsies were compared [12,13,14,15,23,24,25,26,27]. Using the generalized estimating equation method with a logistic regression model, the difference in the major complication ratio between two-week and one-year protocol biopsies, and 95% confidence interval (CI) for the difference in ratio were estimated to confirm that the upper bound of the 95% CI did not exceed 1% of the non-inferiority margin [28,29].

## 3. Results

### 3.1. Baseline Characteristics

There were no significant differences between two-week and one-year protocol biopsies in terms of patient age, sex, body mass index, co-morbidity, history of re-transplantation and delayed graft function, and donor type (Table 1). Pre-biopsy laboratory results were found to be different between the protocol biopsies groups. Two-week protocol biopsy group had lower levels of hemoglobin and activated partial thromboplastin time and higher levels of platelet, blood urea nitrogen, and international normalized ratio compared to late protocol biopsy group. Although international normalized ratio and activated partial thromboplastin time were statistically different between the biopsy groups, they were within the normal range. In addition, the levels of hemoglobin, blood urea nitrogen (BUN), and creatinine are inevitably different between the two-week group and one-year group, because of the effects of preoperative status with chronic kidney disease. SCRs of two-week and one-year protocol biopsies were 15.4% (130/842) and 33.6% (134/399), respectively (*p* < 0.001). Supplementary Table S1 shows the results of detection and treatment according to the SCR type.

### 3.2. Techniques of Protocol Biopsies

The mean number of cores of two-week and one-year protocol biopsies was 2.4 ± 1.0 and 2.5 ± 1.0, respectively (*p* = 0.630) (Table 2). The mean number of glomeruli of the biopsies was 28.8 ± 13.6 and 24.8 ± 12.75, respectively (*p* < 0.001). The core length of two-week protocol biopsy was shorter (*p* < 0.001) and the procedure time was longer than one-year protocol (*p* = 0.007). Although there was significant difference in terms of the number of glomeruli and core length, both protocol biopsies met for technical success. Besides, the mean procedure times were both within 10 min. There was no difference in technical success among radiologists with different experiences (*p* = 1.000). 

### 3.3. Complications of Protocol Biopsies

The major complication of two-week protocol biopsy occurred in three cases (Table 3). They underwent re-operation for bleeding control in one, arterial embolization in one, and transfusion in one case. The major complication of one-year protocol biopsy occurred in 1 case, resulting in graft loss. The major complication rates of two-week and one-year protocol biopsies were 0.3% (3/842) and 0.2% (1/399), respectively. There was no difference in the incidence of major complications among radiologists with different experiences (*p* = 0.453).

### 3.4. Non-Inferiority Test of Protocol Biopsies

The 95% CI for the difference in the ratio between two-week and one-year protocol biopsies were −0.29% and 0.84%, and the upper bound is less than the non-inferiority margin 1% in terms of major complication. The non-inferiority analysis showed that there was no difference between the protocol biopsies. Therefore, the safety of early protocol biopsy was non-inferior to that of late protocol biopsy.

### 3.5. Risk Factors for Major Complications

Univariate analysis showed that low platelet count (<100 K) and high BUN (≥40 mg/dL) were potential risk factors for major complication prior to two-week protocol biopsy (*p* = 0.012 and *p* = 0.009, respectively). The odds ratios of platelet count <100 K and BUN ≥40 mg/dL were 22.806 (1.977–263.082) and 25.065 (2.241–280.284), respectively. However, multivariate analysis was not possible due to insufficient number of major complication cases.

## 4. Discussion

Early protocol biopsy is conducted usually from 1 month to 6 months in many institutes after renal allograft is transplanted [12,13,14,15]. In contrast, most of our initial early protocol biopsies were performed around 2 weeks (median, 12 days), and a substantial number of SCRs were detected in patients who do not have clinical or laboratory findings of acute rejection. Accordingly, many SCRs could be detected and treated in the earlier stage. There are only a few studies reporting the value of early protocol biopsy to determine if it can detect SCR when it is performed within one month [30,31]. Our study demonstrated that post-operative biopsies at 2 weeks are not too early for early protocol biopsy for detecting SCR. 

Technical feasibility and safety are two important issues to concern in conducting two-week protocol biopsy. Experience of our radiologists ranged widely. Some were beginners and others were experts in performing US-guided biopsy. However, there was no difference in terms of technical success and major complication. These findings are suggesting that two-week protocol biopsy is not so difficult procedures. They did not use a biopsy guider, but free hand techniques in sampling core tissues from the cortex. All radiologists obtained adequate cores, in which more than 20 glomeruli were present. Other studies showed that the adequacy rate was 80–99% [32,33], which was not higher than that in our study. As a result, these findings suggest that two-week protocol biopsy as well one-year protocol biopsy is feasible regardless of radiologist experience period.

For two-week protocol biopsy, surgical incision is just healed and fibrosis with surrounding tissue is not still established around the renal allograft. Therefore, early protocol biopsy is susceptible to cause bleeding projecting out from the renal capsule. However, we performed manual compression as a routine procedure following biopsy. Radiologists are easy to detect bleeding using US scan after the compression is finished. If bleeding is still present, manual compression was continued until it disappeared. For these reasons, major bleeding requiring re-operation, embolization, or transfusion was very low following early protocol biopsy.

According to previously reported research results, the major complication rate of early protocol biopsy ranges from 0.4% to 4.3% [13,22,23,24,26,34]. The overall complication rate of this study was 2.1% at the 2-week and 1.0% at the 1-year. Our major complication rate of two-week and one-year protocol biopsy was only 0.3% and 0.2%, respectively. Both biopsies were lower than the report of previous studies. We think that the main reasons why our major complication rates were extremely low compared to those of previous studies include two post-biopsy procedures such as manual compression and US monitoring to determine if there is persistent bleeding. Unfortunately, there was no previous studies dealing with protocol biopsies clearly stated about post-biopsy procedures.

Previous studies have reported that the timing of early protocol biopsy was from 1 month to 6 months [12,13,14,15]. Most of cases of early protocol biopsies in our center were performed around 2 weeks. Only two studies showed that early protocol biopsy was performed postoperative 1–2 weeks after transplantation. However, they did not state about technical feasibility or safety, but just focus on SCR [30,31]. 

We analyzed data on the complications to evaluate the safety of two-week protocol biopsy. The function of the transplanted kidney is normalized about 2 weeks after KT, and the status of the transplanted kidney can be evaluated through the biopsy [30]. In addition, since the patient is discharged after 2 weeks, our early protocol biopsy can be performed during hospitalization. Since the patient is monitored for two or three days before discharge, it is possible to perform safer monitoring. 

Risk factors for major complication of protocol biopsy have been reported in several studies. Morgan et al. demonstrated that increased age, increased BUN, decreased platelet count, history of prior renal transplant, deceased donor transplant type, use of anticoagulant medication but not aspirin were independent risk factors [26]. Redfield et al. reported a decrease in hematocrit or hemoglobin and an increase in post biopsy BUN and creatinine as clinically significant laboratory predictors of complication [23]. Xu et al. reported renal dysfunction and low platelet counts as risk factors for bleeding complications from conventional kidney biopsy [35]. In our study, univariate analysis was performed to identify risk factors for major complication of early protocol biopsy. Low platelet count (<100 K/mL) and elevated BUN (≥40 mg/dL) were identified as risk factors for major complication prior to two-week protocol biopsy. Since the number of major complications was much small, it was impossible to select variables and build multivariable logistic regression model for detecting independent risk factors. 

There are several reports that platelet reduction and increase in BUN may affect complication. Winkelmayer et al. have reported that platelet dysfunction is common in patients with renal dysfunction due to intrinsic platelet abnormalities and impaired platelet-vessel wall interaction [36]. Ferguson et al. have reported an increased risk of complications after kidney biopsy in patients with poor renal function [24]. Uremia is associated with renal function, which is consistent with our findings that an increase in BUN may be a risk factor for a major complication of protocol biopsy. In addition, uremia is known to cause platelet dysfunction and could explain the increase in bleeding complications [23]. Therefore, when performing protocol biopsy at 2 weeks, if BUN ≥ 40 mg/dL, delay biopsy may be considered, and if platelets < 100 K/mL, platelet transfusion may be considered. 

This study has several limitations. First, it was a retrospective study in a single center. Second, multivariate analysis was not performed due to a small number of major complication cases. Third, optimal timing of early protocol biopsy was not determined because the long-term outcome was not evaluated. Further investigation will be necessary how soon it will be performed. Fourth, we used an 18-gauge needle which is not what is recommended in the literature. A 16-gauge has been reported to be the best needle regarding safety and adequacy. As the size of the needle gauge becomes higher, the number of glomeruli is increasing, but the risk of bleeding can be increasing. Post-operative fibrosis of renal allograft is not established when the two-week protocol biopsy is performed. Post-biopsy bleeding can be more excessive when a 16-gauge needle is used. For these reasons, we had to choose an 18-gauge needle for the early protocol biopsy to avoid the risk of massive bleeding, re-operation, or graft failure. However, further investigation is necessary to compare 18-gauge and 16-gauge needles in terms of technical success and post-biopsy bleeding when two-week protocol biopsy is performed. Fifth, a major weakness of this study is the lack of evaluation of the long-term outcomes of the two-week protocol biopsy. Further studies will be conducted on the efficacy of evaluating the long-term outcomes of two-week protocol biopsy.

In conclusion, two-week protocol biopsy can be performed feasibly and safely regardless of radiologist’s experience. However, this early protocol biopsy contributes to detecting a significant number of SCR cases. The major complication rate is as low as that of one-year protocol biopsy. Because low platelet count (<100 K/mL) and elevated BUN (≥40 mg/dL) can be associated with major complication, these laboratory findings should be carefully checked prior to two-week protocol biopsy.

## Figures and Tables

**Table 1:** Baseline characteristics of two-week and one-year protocol biopsies.

Patients Demographics	Two-Week Protocol Biopsy (*n =* 842)	One-Year Protocol Biopsy (*n =* 399)	*p* Value
Age	49.2 ± 11.6	49.9 ± 11.5	0.254
Sex, male	511 (60.7)	248 (62.2)	0.701
BMI	23.0 ± 3.6	23.0 ± 3.5	0.792
Comorbidity			
DM	226 (26.8)	108 (27.1)	0.945
HTN	656 (77.9)	307 (76.9)	0.716
HBV	53 (6.3)	32 (8.0)	0.279
HCV	9 (1.1)	3 (0.8)	0.761
Cardiovascular	52 (6.2)	32 (8.0)	0.276
Cerebrovascular	19 (2.3)	12 (3.0)	0.440
Re-KT	75 (8.9)	34 (8.5)	0.831
Immunological factors			
PRA	105 (12.5)	48 (12.0)	0.854
HLA1 mismatch	297 (35.5)	128 (32.1)	0.277
HLA2 mismatch	200 (23.8)	92 (23.1)	0.830
DSA	116 (13.8)	51 (12.8)	0.657
ABO incompatible	126 (15.0)	61 (15.3)	0.932
DGF	54 (6.4)	29 (7.3)	0.627
Donor type			0.412
Living donor	527 (62.6)	254 (63.7)	
SCD	198 (23.5)	82 (20.6)	
ECD	117 (13.9)	63 (15.8)	
Pre-Bx laboratory result			
Hemoglobin, g/dL	10.0 ± 1.5	12.3 ± 2.0	<0.001
Platelets, /mL	224.2 ± 71.6	204.6 ± 54.4	<0.001
BUN, mg/dL	23.1 ± 12.3	16.5 ± 6.1	<0.001
Serum creatinine, mg/dL	1.35 ± 1.11	1.16 ± 0.38	<0.001
eGFR, mL/min per 1.73 m^2^	66.8 ± 25.8	68.8 ± 19.7	0.142
INR	1.06 ± 0.09	1.02 ± 0.11	<0.001
aPTT	31.7 ± 4.2	36.2 ± 4.1	<0.001
Medication			
Aspirin use	138 (16.4)	78 (19.5)	0.174
Clopidogrel use	15 (1.8)	3 (0.8)	0.206
Timing of biopsy, POD	12 (10–20)	383 (302–420)	<0.001

Data are presented as n (%) or mean (±standard deviation). BMI, body mass index; DM, diabetes mellitus; HTN, hypertension; HBV, hepatitis B virus; HCV, hepatitis C virus; KT, kidney transplantation; DGF, delayed graft function; ECD, expanded criteria donor; SCD, standard criteria donor; BUN, blood urea nitrogen; eGFR, estimated glomerular filtration rate; INR, international normalized ratio; aPTT, activated partial thromboplastin time; POD, postoperative day.

**Table 2:** Comparison of protocol biopsies by technique and outcome.

Variables	Two-Week Protocol Biopsy (*n =* 842)	One-Year Protocol Biopsy (*n =* 399)	*p* Value
Number of cores	2.4 ± 1.0	2.5 ± 1.0	0.630
Number of glomeruli	28.8 ± 13.6	24.8 ± 12.75	<0.001
Length of core (cm)	1.3 ± 0.4	1.4 ± 0.4	<0.001
Procedure time	9 min 32 s ± 4 min 35 s	8 min 49 s ± 4 min 29 s	0.007

Data are presented as mean (±standard deviation).

**Table 3:** Complication rates of protocol biopsies in renal allograft.

Complications, *n* (%)	Two-Week Protocol Biopsy (*n =* 842)	One-Year Protocol Biopsy (*n =* 399)	*p* Value
Major complications	3 (0.3)	1 (0.2)	1.000
Death	0 (0)	0 (0)	
Graft loss	0 (0)	1 (0.2)	
Surgical intervention	1 (0.1)	0	
Radiologic intervention	1 (0.1)	0	
Red blood cell transfusion	1 (0.1)	0	
Minor complications	15 (1.8)	3 (0.7)	0.207
Arteriovenous fistula	4 (0.5)	0	
Minor bleeding	11 (1.3)	3 (0.7)	
New onset gross hematuria	0	0	
Overall complication	18 (2.1)	4 (1.0)	0.178

Data are presented as *n* (%).

## Data Availability

The data are available from the corresponding author upon reasonable request.

## Supplementary Materials

The following supporting information can be downloaded at: https://www.mdpi.com/article/10.3390/jcm11030785/s1, Table S1: Comparison of detection and treatment rates of subclinical rejection between two-week and one-year protocol biopsies.
