# Supplementary material for: Two-Week Protocol Biopsy in Renal Allograft: Feasibility, Safety, and Outcomes
Source: J Clin Med. 2022 Jan 31;11(3):785. doi: 10.3390/jcm11030785 (PMC8836907; doi:10.3390/jcm11030785)
Supplement: Supplementary file 1 [file jcm-11-00785-s001.zip › jcm-1564978-supplementary.pdf]

Supplementary Table S1. Comparison of detection and treatment rates of subclinical rejection between two-week and one-year protocol biopsies

Variables	Two-week protocol Biopsy (*n* = 842)	One-year protocol biopsy (*n* = 399)	P value
Detection			< 0.001
None	712 (84.6 %)	265 (66.4 %)	
SCR	130 (15.4 %)	134 (33.6 %)	
Borderline change	85 (10.1 %)	91 (22.8 %)	
TCMR	28 (3.3 %)	30 (7.5 %)	
AMR	13 (1.5 %)	9 (2.3 %)	
Combined	4 (0.5 %)	4 (1.0 %)	
Treatment rates			0.147
Total	117 / 130 (90.0 %)	112 /134 (83.6 %)	
Borderline change	75 / 85 (88.2 %)	77 / 91 (84.6 %)	
TCMR	28 / 28 (100 %),	23 / 30 (76.7 %)	
AMR	10 / 13 (76.9 %)	8 / 9 (88.9 %)	
Combined	4 / 4 (100 %)	4 / 4 (100 %)	

Data are presented as *n* (%).

SCR, subclinical rejection; TCMR, T-cell mediated rejection; AMR, antibody-mediated rejection.
